# The Monetite Structure Probed by Advanced Solid-State NMR Experimentation at Fast Magic-Angle Spinning

**DOI:** 10.3390/ijms20246356

**Published:** 2019-12-17

**Authors:** Yang Yu, Baltzar Stevensson, Michael Pujari-Palmer, Hua Guo, Håkan Engqvist, Mattias Edén

**Affiliations:** 1 Department of Materials and Environmental Chemistry, Stockholm University, SE-106 91 Stockholm, Sweden; yang.yu@mmk.su.se (Y.Y.); baltzar.stevensson@mmk.su.se (B.S.); hua.guo@mmk.su.se (H.G.); 2 Applied Material Science, Department of Engineering, Uppsala University, SE-751 21 Uppsala, Sweden; michael.palmer@angstrom.uu.se (M.P.-P.); hakan.engqvist@angstrom.uu.se (H.E.)

**Keywords:** monetite structure, calcium phosphate cement, bioceramics, internuclear distance determination, double-quantum correlation 1H NMR, ^31^P{1H} HETCOR NMR

## Abstract

We present a solid-state nuclear magnetic resonance (NMR) spectroscopy study of the local ${}^{31}$P and ${}^{1}$H environments in monetite [CaHPO${}_{4}$; dicalcium phosphate anhydrous (DCPA)], as well as their relative spatial proximities. Each of the three ${}^{1}$H NMR peaks was unambiguously assigned to its respective crystallographically unique H site of monetite, while their pairwise spatial proximities were probed by homonuclear ${}^{1}$H–${}^{1}$H double quantum–single quantum NMR experimentation under fast magic-angle spinning (MAS) of 66 kHz. We also examined the relative ${}^{1}$H–${}^{31}$P proximities among the inequivalent {P1, P2} and {H1, H2, H3} sites in monetite; the corresponding shortest internuclear ${}^{1}$H–${}^{31}$P distances accorded well with those of a previous neutron diffraction study. The NMR results from the monetite phase were also contrasted with those observed from the monetite component present in a pyrophosphate-bearing calcium phosphate cement, demonstrating that while the latter represents a disordered form of monetite, it shares all essential local features of the monetite structure.

## 1. Introduction

The natural mineral monetite (CaHPO${}_{4}$), also referred to as dicalcium phosphate anhydrous (DCPA), is the anhydrous form of brushite (CaHPO${}_{4}\cdot$2H${}_{2}$O; dicalcium phosphate dihydrate; DCPD). The latter is of interest for biomineralization, both as a potential precursor phase of bone mineral [1], as well as its appearance under acidic conditions associated with pathological bone mineralization pathways that for instance lead to dental calculus and kidney stone [2]. Both monetite and brushite are also main constituents in calcium phosphate cement (CPC) formulations used as biomedical implants in dental, craniofacial, and orthopedic surgeries [3,4,5].

**Figure 1:**
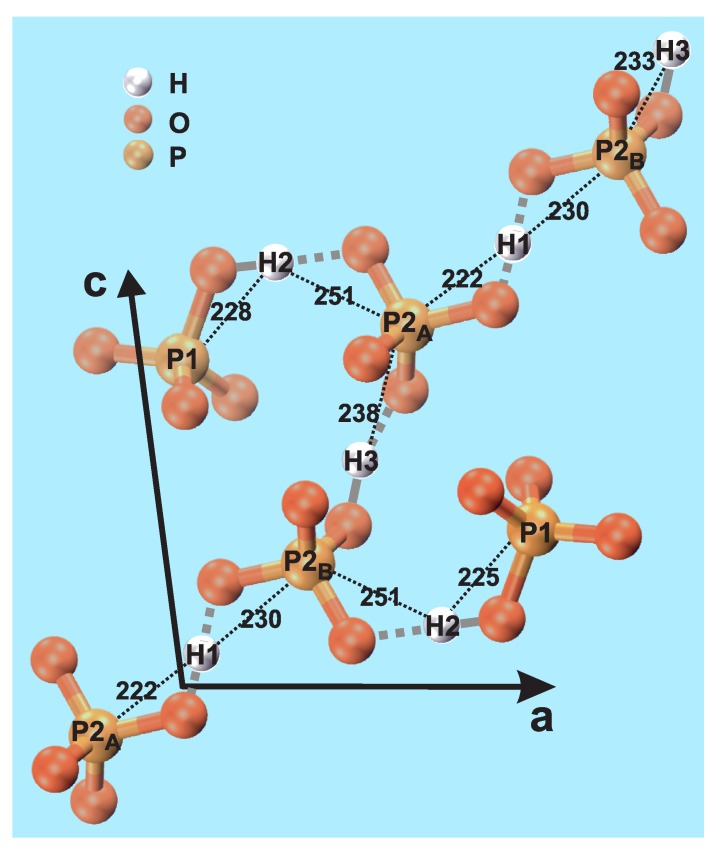
Structural fragment of monetite (CaHPO${}_{4}$; DCPA) [6], indicating each of the two crystallographically unique P1 and P2 sites (where P2${}_{A}$ and P2${}_{B}$ differ in their H1/H3 proton neighbors), as well as the three inequivalent H1, H2, and H3 sites. The black dotted lines indicate the interatomic H–P distances (in pm). The corresponding O–H contacts are highlighted by grey lines, where a solid line connects the (acidic) proton of an HPO${}_{4}^{2-}$ moiety, whereas each dotted line marks an H bond (H⋯O). Note that there is a “chain” of HPO${}_{4}^{2-}$ tetrahedra that involves P2${}_{A}$ and P2${}_{B}$ sites alternating with H3 and H1 protons to form a H-bonded network. We stress that the covalent/H-bond classification of P–O–H and P–O⋯H is somewhat simplified.

Monetite exists in two modifications that only differ in their H environments, where the “high-temperature” form (space group $P\bar{1}$) is stable at room temperature; a fragment of the latter structure is shown in Figure 1, as obtained by Catti et al. [6] using neutron diffraction. The structure comprises three unique H sites; H1, H2, and H3 with multiplicity 1:2:1, along with two crystallographically inequivalent P sites: P1 and P2. However, the P2 sites may be separated into P2${}_{A}$ and P2${}_{B}$ that feature distinct H environments; see Figure 1. The P1:P2${}_{A}$:P2${}_{B}$ multiplicities are 2:1:1, meaning that P1:P2 exhibit equal multiplicities [6].

Besides X-ray and neutron diffraction studies [6,7,8,9,10,11], magic-angle-spinning (MAS) nuclear magnetic resonance (NMR) has been utilized to probe the *local* monetite structure [12,13,14,15,16,17,18,19,20]. ${}^{31}$P MAS NMR readily resolves the resonances from the distinct P1 and P2 sites [12,13,15,16,17,18,20]. Yet, while the spectral resolution is insufficient for discriminating between the two P2${}_{A}$ and P2${}_{B}$ environments, their presence are evident from the markedly broader ${}^{31}$P2 resonances relative to their ${}^{31}$P1 counterparts [15,18,20], as discussed further herein. The inequivalent H1, H2, and H3 sites of monetite have also been examined by ${}^{1}$H MAS NMR experimentation [14,19,20]. However, while even low MAS rates of <15 kHz readily discriminate the ${}^{1}$H1 resonance from its ${}^{1}$H2/${}^{1}$H3 counterparts [14], the close chemical shifts of the latter coupled with resonance-broadenings from ${}^{1}$H–${}^{1}$H dipolar interactions (see below) and structural disorder of the H3 sites [6,12] may prevent their clear discrimination. Here we provide the first unambiguous ${}^{1}$H NMR-peak assignments to the three proton sites in monetite; they confirm those given previously by Xue and Kanzaki based on an NMR spectrum with heavily overlapping ${}^{1}$H2/${}^{1}$H3 signals [19].

The ${}^{1}$H MAS NMR spectrum alone, however, does not reveal the relative spatial positions of the various proton sites in a structure. Such information may be gathered from more advanced solid-state NMR experimentation that relies on the ${}^{1}$H–${}^{1}$H dipolar interaction, which is mediated directly *through space* (in contrast with the through-*bond* *J* interactions frequently utilized in solution NMR). The interaction strength is given by the dipolar coupling constant ($b_{\mathrm{HH}}$; units of Hz), which depends on the inverse cube of the ${}^{1}$H–${}^{1}$H internuclear distance ($r_{\mathrm{HH}}$) [21,22,23,24,25]. Hence, these NMR interactions may reveal pair-wise spatial proton proximities. Although the typically large ${}^{1}$H–${}^{1}$H dipolar interactions are incompletely suppressed even at very fast MAS, and thereby limit the resolution in MAS NMR spectra [21,22], their utilization for obtaining qualitative/quantitative interatomic-distance information requires application of *dipolar recoupling* rf-pulse sequences to restore (i.e., “recouple”) the dipolar effects in a controlled fashion under fast MAS conditions [21,22,23,24]. In this work, we employed the symmetry-based [24] recoupling schemes [*S*R2${}_{2}^{1}$] and [*S*R2${}_{4}^{1}$] [26,27,28,29,30] to excite ${}^{1}$H double-quantum (2Q) coherences (2QC) that provide internuclear-distance information within pairs of interacting protons [21,22,24]. These rf-pulse schemes were originally introduced for 2QC excitation among half-integer spins [26,27,30], but has also been utilized for probing ${}^{1}$H–${}^{1}$H proximities at the surface of mesoporous silica [31], as well as for studying pyrophosphate-rich monetite-based cements [32]; the latter experimentation involved double-quantum-single-quantum (2Q–1Q) correlation ${}^{31}$P NMR experiments aiming at improving the understanding of the structural role of the various amorphous and crystalline bioactive pyrophosphate species, which have been demonstrated to stimulate bone growth [33].

Herein, we evaluate what information correlation NMR experiments targeting ${}^{1}$H–${}^{1}$H and ${}^{1}$H–${}^{31}$P proximities may provide about the monetite structure. We report 2Q–1Q correlation ${}^{1}$H NMR experimentation on a monetite reference sample, “*Mon*”, as well as on the disordered “monetite-like” phase present in a CPC that incorporates 15 wt% P${}_{2}$O${}_{7}^{4-}$. The latter specimen is henceforth referred to as “*MonCPC*”. It was characterized by complementary NMR correlation experiments in ref. [32]. Furthermore, we probe the relative ${}^{1}$H–${}^{31}$P proximities among the inequivalent {P1, P2} and {H1, H2, H3} sites in the monetite structure using ${}^{31}$P{${}^{1}$H} heteronuclear correlation (HETCOR) NMR [34]. The thereby determined “effective” (average) H–P distances agreed very well with their neutron diffraction-derived counterparts (Figure 1). The HETCOR results are also discussed in relation to those reported previously on the MonCPC cement [32].

## 2. Materials and Methods

### 2.1. Samples

The monetite sample (“Mon”) was prepared from brushite purchased from Sigma-Aldrich, Munich, Germany (98% purity) by heating at 140 ${}^{\circ }$C and 2.5 bar in an autoclave for 1 h (CertoClav EL, CertoClav Sterilizer, GmbH, Traun, Austria). It was subsequently heated at 120 ${}^{\circ }$C for 4 h prior to the NMR experimentation. The MonCPC cement was prepared by mixing powders of 565 mg β-Ca${}_{3}($PO${}_{4}$)${}_{2}$ (<0.5 μm particles; Sigma-Aldrich), 435 mg Ca(H${}_{2}$PO${}_{4}$)${}_{2}\cdot$2H${}_{2}$O (25–50 μm particles; 98% purity; Scharlau, Barcelona, Spain), and 176.4 mg of Na${}_{2}$H${}_{2}$P${}_{2}$O${}_{7}$ (99%; Sigma-Aldrich). The β-TCP powder comprised 9.14 wt% of β-Ca${}_{2}$P${}_{2}$O${}_{7}$. Premixed β-TCP and Na${}_{2}$H${}_{2}$P${}_{2}$O${}_{7}$ powders were mixed with Ca(H${}_{2}$PO${}_{4}$)${}_{2}\cdot$2H${}_{2}$O in a turbula for 15 min (Turbula Schatz T2F; Eskens Group BV, Rijn, The Netherlands), whereupon a 0.5 M aqueous solution of citric acid was added (liquid to powder ratio of 0.25 mL/g), followed by further mixing by vortex for 30 s in a cap shaker for 60 s (ESPE Capmix; 3M ESPE AG, Seefeld, Germany). The cement was then allowed to set for 72 h in a sealed container at 37 ${}^{\circ }$C and 100% humidity, followed by autoclaving for 1 h at 140 ${}^{\circ }$C and 2.5 bar to convert the as-formed brushite phase into (mainly) monetite.

### 2.2. Solid-State NMR

All solid-state NMR experimentation was performed with Bruker Avance-III spectrometers (Bruker BioSpin; Rheinstetten, Germany) and magnetic fields of 9.4 T and 14.1 T that provided ${}^{1}$H/${}^{31}$P Larmor frequencies of −400.1/−162.0 MHz and −600.1/−242.9 MHz, respectively. Fine powders of the “Mon” and “MonCPC” samples were packed in ZrO${}_{2}$ rotors with outer diameters of 1.3 mm, 2.5 mm (“thin wall”), or 4 mm, which were spun at MAS rates ($\nu_{r}$) of 66.00 kHz, 34.00 kHz, and 14.00 kHz, respectively. ${}^{1}$H and ${}^{31}$P chemical shifts are quoted relative to neat tetramethylsilane (TMS) and 85% H${}_{3}$PO${}_{4}$(aq), respectively. The uncertainty of each reported chemical shift was estimated as ±0.1 ppm for both ${}^{1}$H and ${}^{31}$P. Yet, we note that the NMR peak positions among independent ${}^{1}$H and ${}^{31}$P NMR experiments are reproducible well within the uncertainty span of 0.2 ppm.

Resonance offsets were minimized by positioning the radio-frequency (rf) carrier (“transmitter”) ${}^{1}$H/${}^{31}$P frequency at the mid of the NMR signal region throughout all NMR experiments. To accomplish absorptive 2D NMR peaks with frequency-sign discrimination along the indirect spectral dimension, all 2D NMR acquisitions implemented the States-TPPI procedure [35]. Note that each number of $t_{1}$ increments stated below refers to that collected for *each* real/imaginary data-set of the hypercomplex protocol.

#### 2.2.1. Single-Pulse NMR Experiments

The single-pulse (“Bloch decay”) ${}^{31}$P NMR spectrum recorded from the standard Mon reference sample at $B_{0}=9.4$ T and $\nu_{r}=14.00$ kHz utilized 90${}^{\circ }$ rf excitation pulses operating at the ${}^{31}$P nutation frequency $\nu_{P}=87$ kHz, 4 accumulated signal transients, and 40 s relaxation delays. Throughout the ${}^{31}$P NMR signal detection, the SPINAL-64 rf-pulse sequence [36] with 5.4 μs pulses operating at the ${}^{1}$H nutation frequency $\nu_{H}=80$ kHz was used for proton decoupling.

Single-pulse ${}^{1}$H NMR spectra were collected from the monetite standard using 90${}^{\circ }$ excitation pulses, 5 s relaxation delays, along with the following conditions: $B_{0}=9.4$ T, $\nu_{r}=14.00$ kHz (4 mm rotor), $\nu_{H}=80$ kHz, and 16 accumulated signal transients; $B_{0}=9.4$ T, $\nu_{r}=34.00$ kHz (2.5 mm rotor), $\nu_{H}=102$ kHz, and 128 transients; $B_{0}=14.1$ T, $\nu_{r}=66.00$ kHz (1.3 mm rotor), $\nu_{H}=139$ kHz, and 64 transients. The presented NMR spectra recorded with 2.5 mm and 1.3 mm rotors were corrected for “background” ${}^{1}$H signals by subtracting the result obtained from an empty rotor under otherwise identical experimental conditions.

The ${}^{31}$P ($\nu_{r}=14.00$ kHz) and ${}^{1}$H NMR ($\nu_{r}=66.00$ kHz) spectra were deconvoluted using a MatLab program developed in our laboratory [37,38]. It minimizes the $\chi^{2}$-deviation between the experimental and calculated NMR spectra, while permitting both free and constrained parameter ranges. The ${}^{1}$H NMR spectrum was deconvoluted by using one peak from each {H1, H2, H3} site, employing Lorentzian peakshapes for H1 and H2, and a mixed Gaussian/Lorentzian shape for H3. The chemical shifts (“peak positions”) were allowed to vary freely, while the full with at half maximum (FWHM) height of each NMR peak was constrained as FWHM ⩾ 0.7 ppm. The ${}^{31}$P NMR spectrum was deconvoluted into resonances from ${}^{31}$P1 and ${}^{31}$P2, respectively. However, as discussed further in refs. [32,39] and the caption to Figure S1 of the Supporting Information, *each* ${}^{31}$P1/${}^{31}$P2 resonance were emulated by *two* NMR peak components. These two components were invoked to account for the structural disorder, whereas no attempts were made to further separate the two (heavily) overlapping ${}^{31}$P2${}_{A}$ and ${}^{31}$P2${}_{B}$ resonances (see Section 3.1 and Section 3.4.3).

#### 2.2.2. 2Q–1Q ${}^{1}$H NMR Experiments

Two 2Q–1Q ${}^{1}$H correlation spectra were recorded from the Mon specimen at $B_{0}=14.1$ T and $\nu_{r}=66.00$ kHz, using the 2D NMR protocol shown in Figure 1c of ref. [29], except that 2QC were generated directly from longitudinal ${}^{1}$H polarization. Either one completed [*S*R2${}_{2}^{1}$] sequence ($\tau_{\mathrm{exc}}=\tau_{\mathrm{rec}}=60.6$ μs) or two completed [*S*R2${}_{4}^{1}$] sequences ($\tau_{\mathrm{exc}}=\tau_{\mathrm{rec}}=242.4$ μs) were utilized for 2QC excitation/reconversion [26,27,28,29]; the brackets [⋯] imply sandwiching each *S*R2${}_{2}^{1}\equiv$R2${}_{2}^{1}$R2${}_{2}^{-1}$ or *S*R2${}_{4}^{1}\equiv$R2${}_{4}^{1}$R2${}_{4}^{-1}$ pulse sequence by two strong 90${}^{\circ }$ pulses [26,27,28,29]. The ${}^{1}$H nutation frequency was $\nu_{H}=\nu_{r}/2=33.0$ kHz during dipolar recoupling and 139 kHz for all strong 90${}^{\circ }$/180${}^{\circ }$ pulses. A spin-echo of duration of $2\tau_{r}=30.3$ μs was used prior to the $t_{1}$-evolution interval, where $\tau_{r}=$$\nu_{r}^{-1}$ is the rotor period. For both 2D NMR experiments, 40$\left(t_{1}\right)$ × 2624$\left(t_{2}\right)$ time-points were acquired with dwell times of {$\Delta t_{1}=6\tau_{r}$; $\Delta t_{2}=7.6\;\mu$s}, relaxation delays of 1.0 s, along with 128 and 256 accumulated transients/$t_{1}$-value for the acquisition with $\tau_{\mathrm{exc}}=60.6$ μs and $\tau_{\mathrm{exc}}=242.4$ μs, respectively. Each 2D data set was zero-filled to 256 × 16,384 time points.

Similar 2Q–1Q correlation ${}^{1}$H NMR experiments were performed on the MonCPC sample at $B_{0}=9.4\;$T and $\nu_{r}=34.00$ kHz. The ${}^{1}$H nutation frequency was $\nu_{H}=\nu_{r}/2=17.0$ kHz during dipolar recoupling and 111 kHz for all strong 90${}^{\circ }$/180${}^{\circ }$ pulses. The spin-echo period before the $t_{1}$-evolution interval was $2\tau_{r}=58.8$ μs. 35$\left(t_{1}\right)$ × 1500$\left(t_{2}\right)$ time-points were acquired with 256 transients/$t_{1}$-value and dwell times of {$\Delta t_{1}=2\tau_{r}$; $\Delta t_{2}=9.8\;\mu$s}, using 1.5 s relaxation delays and zero-filling to 256 × 8192 points.

#### 2.2.3. ${}^{31}$P{${}^{1}$H} HETCOR NMR Experiments

Two ${}^{31}$P{${}^{1}$H} HETCOR NMR spectra were recorded from the Mon sample at $B_{0}=14.1\;$T and $\nu_{r}=66.00\;$kHz, using the double-quantum Hartmann-Hahn condition, $\nu_{H}+\nu_{P}=\nu_{r}$, during ${}^{1}$H$\to^{31}$P cross polarization (CP), with $\nu_{H}=44\;$kHz and $\nu_{P}$ ramped by ±2kHz around $\nu_{P}=20\;$kHz. No ${}^{1}$H decoupling was employed during the ${}^{31}$P NMR signal acquisition. For the HETCOR NMR experiment with a short contact period ($\tau_{\mathrm{CP}}$) of 75.8 μs, 14$\left(t_{1}\right)$ × 1970$\left(t_{2}\right)$ data points were collected with 576 accumulated signal transients per $t_{1}$-value, whereas for the acquisition with $\tau_{\mathrm{CP}}=500.0$ μs, 18$\left(t_{1}\right)$ × 1970$\left(t_{2}\right)$ data points were recorded with 192 transients/$t_{1}$-value. Both 2D NMR acquisitions employed dwell times of {$\Delta t_{1}=22\tau_{r}$; $\Delta t_{2}=15.2\;\mu$s}, 1.5 s relaxation delays, with each data-set zero-filled to 256 × 8192 points.

## 3. Results and Discussion

### 3.1. Local ${}^{31}$P and ${}^{1}$H Environments: MAS NMR Results

**Figure 2:**
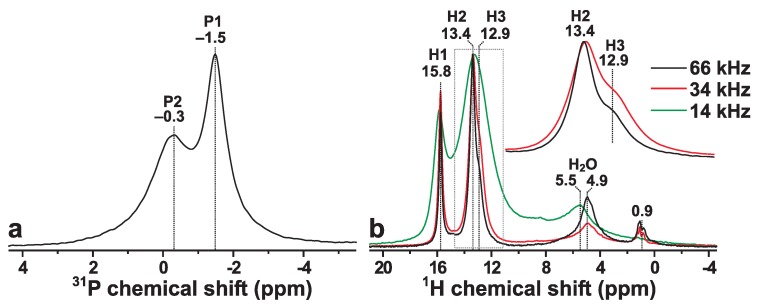
(**a**) ${}^{31}$P NMR spectrum recorded at 14.00 kHz MAS from a powder of monetite (raw data reproduced from Yu et al. [32]). (**b**) ${}^{1}$H MAS NMR spectra recorded from monetite for elevating MAS rates between 14.00 kHz and 66.00 kHz. The inset spectra in (**b**) are zooms around the spectral region indicated by the dotted rectangle. The progressive peak-narrowing for increasing spinning speed stems from the suppression of broadenings from ${}^{1}$H–${}^{1}$H dipolar interactions, which at the higher rates readily resolve the resonances from the two crystallographically inequivalent H2 (13.4 ppm) and H3 (12.9 ppm) sites, whereas the NMR signal from the H1 counterpart (15.8 ppm) is well-separated from the H2/H3 resonances at all MAS rates. Note that the ${}^{1}$H MAS NMR spectrum recorded at 66.00 kHz MAS was obtained at $B_{0}=14.1$ T, whereas all other ${}^{31}$P and ${}^{1}$H NMR spectra were acquired at $B_{0}=9.4$ T.

The presence of two crystallographically inequivalent P sites of monetite (P1 and P2; see Figure 1) is reflected by the ${}^{31}$P MAS NMR spectrum recorded from the Mon sample shown in Figure 2a. The spectrum reveals two ${}^{31}$P resonances at −0.3 ppm and −1.5 ppm, which are associated with the P2 and P1 sites of HPO${}_{4}^{2-}$ groups, respectively. Both signals are relatively broad due to structural disorder [6,12], which particularly concerns the peak at −0.3 ppm that has contributions from two overlapping signals from the P2${}_{A}$ and P2${}_{B}$ sites, which are distinguished by their distinct distances to their proton neighbors (see Figure 1). The relative integrated NMR-signal ${}^{31}$P1:${}^{31}$P2 intensities obtained by deconvoluting the ${}^{31}$P NMR spectrum of Figure 2a are 1.1:1.0, in good agreement with the crystal structure of monetite [6]. The best-fit results are shown in Figure S1 of the Supporting Information.

Figure 2b displays the corresponding ${}^{1}$H NMR spectra observed from the Mon sample at increasing MAS rates between 14.00 kHz and 66.00 kHz. All NMR peaks appearing in the range ≳9 ppm stem from the acidic protons of the HPO${}_{4}^{2-}$ groups [14,19,20], whereas the 4–8 ppm spectral region reveals broad signals from physisorbed water molecules [20]. Furthermore, the presence of minute surface-associated OH groups are suggested by the narrow NMR peaks ≈1 ppm. Onwards, we only consider the high-ppm region (≳9 ppm), as all other NMR signals vanish after the 2QC ${}^{1}$H excitation and reconversion stages (see Section 3.2), while moreover no ${}^{1}$H$\to^{31}$P magnetization transfers were observed from the ${}^{1}$**H**${}_{2}$O/O${}^{1}$**H** sites (Section 3.4).

Owing to a progressive suppression of broadenings from ${}^{1}$H–${}^{1}$H dipolar interactions, Figure 2b evidences markedly narrower NMR peaks when the MAS rate is increased from 14.00 kHz to 66.00 kHz. Indeed, higher MAS rates ⩾34 kHz were sufficient to resolve the NMR responses from the two crystallographically inequivalent H2 (13.4 ppm) and H3 (12.9 ppm) sites of multiplicity 2:1. Deconvolution of the ${}^{1}$H NMR spectrum obtained at 66.00 kHz (Figure S1) yielded the relative abundances of 1.0:2.4:1.0 for the H1:H2:H3 sites, in reasonable agreement with the structure reported using neutron diffraction [6]. Hence, our NMR-peak assignments in Figure 2 confirm the tentative assignments made by Xue and Kanzaki [19]: their ${}^{1}$H MAS NMR spectrum obtained at 40 kHz MAS and the same magnetic field ($B_{0}=9.4$ T) as ours (Figure 2b; 34 kHz) revealed a markedly worse resolution than that of Figure 2b, with the ${}^{1}$H3 signal merely being *hinted* as a “shoulder”/“tail” of the narrower ${}^{1}$H2 NMR peak [19]. Our ${}^{1}$H MAS NMR spectrum accords well with that presented by Pourpoint et al. [40] using the same MAS rate and magnetic field. However, Pourpoint et al. did not provide any NMR-peak assignments. We conclude that the ${}^{1}$H chemical shifts observed herein accord very well with theirs, as well as with those deduced by Xue and Kanzaki [19] at {15.9, 13.5, 13.0} ppm for the respective {H1, H2, H3} sites.

### 3.2. ${}^{1}$H–${}^{1}$H Proximities in Monetite: 2Q–1Q Correlation NMR

**Figure 3:**
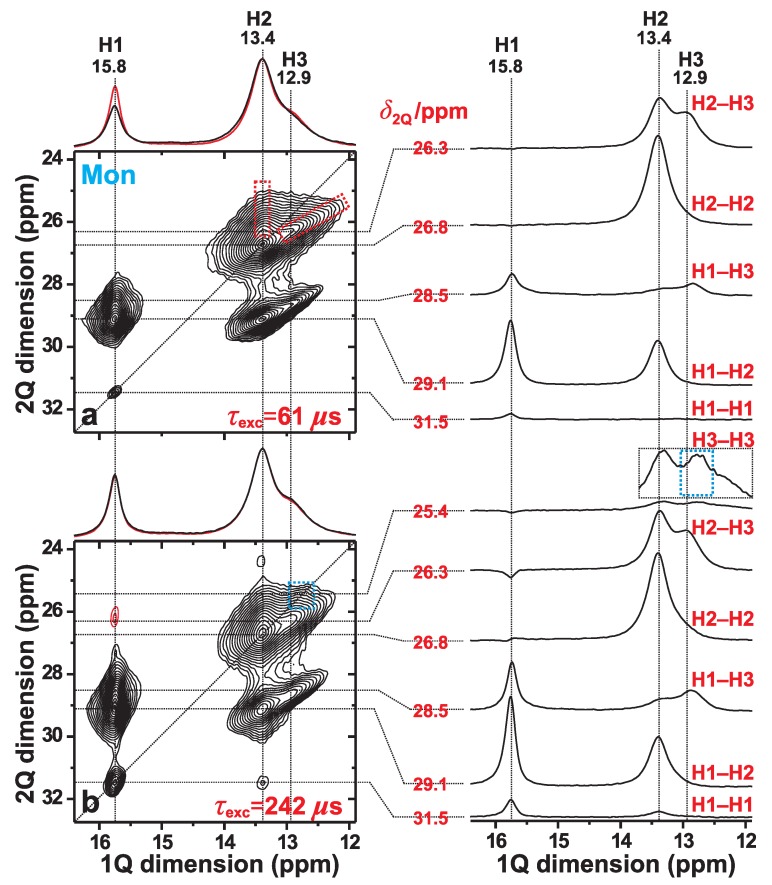
2Q–1Q correlation ${}^{1}$H NMR spectra (left panel) acquired from monetite at 66.00 kHz MAS and $B_{0}=14.1$ T. The (**a**) [*S*R2${}_{2}^{1}$] (ref. [26]) and (**b**) [*S*R2${}_{4}^{1}$] (ref. [27]) pulse sequences were employed for 2QC excitation periods of (**a**) 61 μs and (**b**) 242 μs. The horizontal 1Q projection is shown at the top of the 2D NMR spectrum (black trace), along with the corresponding MAS NMR spectrum (red trace). The NMR-peak assignments to the inequivalent H1, H2, and H3 sites of monetite are indicated at the top of each 2D NMR spectrum. The right panel displays slices along the 1Q dimension, extracted at the as-indicated 2Q shifts ($\delta_{2Q}$) for the 2Q(H*m*–H*n*) correlations identified to the right of each slice. The dashed red rectangles in (**a**) indicates the signal regions associated with the ${}^{1}$H2–${}^{1}$H3 correlations, while the blue rectangle in (**b**) highlights the ${}^{1}$H3–${}^{1}$H3 auto-correlation ridge that emerges at longer excitation periods. The lowest contour level is set at 5% of the maximum 2D NMR peak amplitude, with red contours indicating (minor) negative signal intensities.

We now focus on the spatial proximities among the H1, H2, and H3 proton sites of monetite, as probed by 2Q–1Q correlation ${}^{1}$H NMR at fast MAS of 66 kHz. We first consider the 2D NMR spectrum of Figure 3a, which was recorded from the monetite sample (Mon) by using a short 2QC excitation period of $\tau_{\mathrm{exc}}=61$ μs, thereby only revealing signals from nearest-neighboring ${}^{1}$H sites. In such a 2Q–1Q correlation NMR spectrum, a close proximity between two ${}^{1}$H*m* and ${}^{1}$H*n* sites that resonate at the respective shifts $\delta_{H}^{m}$ and $\delta_{H}^{n}$ along the horizontal (“direct”) “1Q dimension” ($\delta_{1Q}$) is evidenced by two 2D NMR peaks appearing at the coordinates {$\delta_{2Q}^{mn}$, $\delta_{H}^{m}$} and {$\delta_{2Q}^{mn}$, $\delta_{H}^{n}$}. Here the 2QC shift, $\delta_{2Q}^{mn}=\delta_{H}^{m}+\delta_{H}^{n}$, appears along the vertical (“indirect”) “2Q dimension” ($\delta_{2Q}$) of the 2D NMR spectrum [21,41].

However, while spatial proximities among crystallographically distinct proton sites produce two NMR peaks per 2Q–1Q correlation, two nearby *equivalent* protons (e.g., H*m*–H*m*) only give *one* 2D NMR peak, which appear at the 2D NMR coordinate {$\delta_{2Q}$, $\delta_{1Q}$} = {2$\delta_{H}^{m}$, $\delta_{H}^{m}$}. Such “autocorrelation” peaks align along the “diagonal” of the spectrum, whose direction is indicated by the dotted line of slope 2 in Figure 3a. The overall most intense signal at {$\delta_{2Q}$, $\delta_{1Q}$} = {26.8, 13.4} ppm of the 2Q–1Q correlation NMR spectrum stems from the “autocorrelation” of the H2 sites in the monetite structure. This is consistent with the neutron-diffraction-derived structure of ref. [6], whose shortest ${}^{1}$H–${}^{1}$H distances are listed in Table 1: the *overall* closest H–H contact involves H2–H2 (separated by 323 pm), along with another (short) interatomic distance of 357 pm for that proton-pair [6]. Here and onwards, a close/strong “contact” implies a short H*m*–H*n* interatomic distance and/or several protons in close proximity.

Besides the H2–H2 autocorrelation peak, the 2D NMR spectrum of Figure 3a is dominated by two intense pairs of 2QC-correlation “ridges”. They extend between the $\delta_{2Q}$ shift-ranges of 28–30 ppm and 25–27 ppm and originate from the H1–H2 and H2–H3 proton pairs, respectively. Such 2D NMR “ridges” arise from the relatively broad ${}^{1}$H resonances, as is particularly evident for all 2Q–1Q NMR correlation signals involving the H3 sites. Note that contributions from ${}^{1}$H1–${}^{1}$H3 correlations, which overlap with those of ${}^{1}$H1–${}^{1}$H2, account mainly for the extension of the right 2D NMR-signal ridge towards lower ${}^{1}$H chemical shifts along the 1Q dimension of the 2Q–1Q NMR spectrum in Figure 3a. As expected from the relatively long distance of 435 pm between the closest H1–H3 neighbors (Table 1), the ${}^{1}$H1–${}^{1}$H3 signal intensities are comparatively weak relative to their ${}^{1}$H1–${}^{1}$H2 counterparts, as may be verified from the slices along the 1Q dimension of the 2D NMR spectrum shown in the right panel of Figure 3a.

For short 2QC excitation periods—such as that of $\tau_{\mathrm{exc}}=61$ μs employed to record the 2Q–1Q NMR spectrum of Figure 3a—the integrated 2D NMR peak intensity [I(H*m*–Hn)] stemming from a proton pair ${}^{1}$H*m*–${}^{1}$H*n* is proportional to $b^{2}$(H*m*–H*n*), i.e., to [*r*(H*m*–H*n*)]${}^{-6}$ [21,41]. Yet, the *number* of ${}^{1}$H*m*–${}^{1}$H*n* pairs must also be considered: for a “dipolar-coupling-multiplicity” of *M* of a ${}^{1}$H*m*–${}^{1}$H*n* pair, its squared “effective coupling constant” becomes $b_{\mathrm{eff}}^{2}($H*m*–H$n)=Mb^{2}$(H*m*–H*n*) if all *M* distances are equal, whereas for the case of (slightly) different distances,

(1) $$b_{\mathrm{eff}}^{2}\left(Hm-Hn\right)=\sum_{j=1}^{M}b^{2}\left(H^{j}m{-H}^{j}n\right).$$

Owing to the strong signal overlap between the various 2Q–1Q NMR correlation peaks associated with H2 and H3, the spectral resolution in Figure 3a did not permit analysis of the individual resonances from all six H*m*–H*n* ({*m*, *n*}={1, 2, 3}) pairs. However, a consistency check against the H positions of the crystal structure reported in ref. [6] is possible if the H2 and H3 structural sites are grouped together (“H23”). Then the integrated 2Q–1Q NMR signal intensities from the H1–H1, H1–H23, and H23–H23 pairs provided the respective set of *fractional* signal intensities 0.01:0.38:0.61, where f(H*m*–Hn)=I(H*m*–H$n)/I_{\mathrm{tot}}$, with $I_{\mathrm{tot}}=I($H1–H1)+I(H1–H23)+I(H23–H23). These values are in excellent agreement—well within the experimental uncertainties—with those of 0.00:0.42:0.58 that were calculated from the proton coordinates in ref. [6] and obtained as the ratios $b_{\mathrm{eff}}^{2}($H1–H1$)/b_{\mathrm{eff}}^{2}($tot), $b_{\mathrm{eff}}^{2}($H1–H23$)/b_{\mathrm{eff}}^{2}($tot), and $b_{\mathrm{eff}}^{2}($H23–H23$)/b_{\mathrm{eff}}^{2}($tot), respectively. Here $b_{\mathrm{eff}}^{2}($tot) is the sum over all squared effective dipolar coupling constants [Equation (1)].

Lengthening of the 2QC excitation period enables the probing of progressively longer internuclear ${}^{1}$H–${}^{1}$H distances, i.e., those associated with *smaller* ${}^{1}$H–${}^{1}$H dipolar-coupling constants. Indeed, the 2Q–1Q NMR spectrum of Figure 3b, which was acquired with a 2QC excitation interval of 242 μs, reveals a markedly more intense ${}^{1}$H1–${}^{1}$H1 correlation signal at the diagonal of the 2D NMR spectrum; also compare the ${}^{1}$H1 signal intensity in each slice along the 1Q dimension extracted at $\delta_{2Q}=31.5$ ppm in Figure 3a,b. Moreover, the 2D NMR spectrum in Figure 3b evidences a broad signal-ridge extending along the low-ppm region of the diagonal (marked by the blue rectangle): it emerges for longer excitation intervals and originates from 2QC generation among the more distant H3–H3 pairs. Note that the shortest interatomic distances associated with the H1–H1 and H3–H3 pairs are equal (663 pm) and roughly twice those of their H1–H2 and H2–H2 counterparts; see Table 1. We conclude that the 2Q–1Q NMR results are in very good agreement with the crystal structure reported for monetite [6], while moreover also corroborating the ${}^{1}$H NMR-peak assignments given in ref. [19].

Besides the weak ${}^{1}$H1–${}^{1}$H1 and ${}^{1}$H3–${}^{1}$H3 correlations, the 2D NMR spectrum of Figure 3b also manifests a minor peak at the 2D NMR coordinate $\{\delta_{2Q}$, $\delta_{1Q}\}=\{$31.5, 13.4} ppm. This signal reflects a 2QC correlation among two H1 protons in the indirect spectral dimension; *yet*, the magnetization ended up at the H2 site during the ${}^{1}$H 1Q NMR signal detection. Such an exotic “indirect 2QC signal” [41] occurs from the presence of a strong ${}^{1}$H1–${}^{1}$H2 dipolar interaction, as discussed further in refs. [21,41]. It is analogous to “relayed transfers” in homonuclear magnetization-transfer NMR experiments [22,23]. Such indirect 2QC correlations also account for the weak *negative* NMR signal amplitudes observed at $\delta_{1Q}\approx 16$ ppm in the 1Q dimension of the 2Q–1Q correlation NMR spectrum of Figure 3b that extend along $\delta_{2Q}$ shift-range of 25–27 ppm (see the slices along the 1Q dimension). Those NMR correlation signals are associated with the H1 site.

### 3.3. ${}^{1}$H–${}^{1}$H Proximities in the Monetite-Based Cement

**Figure 4:**
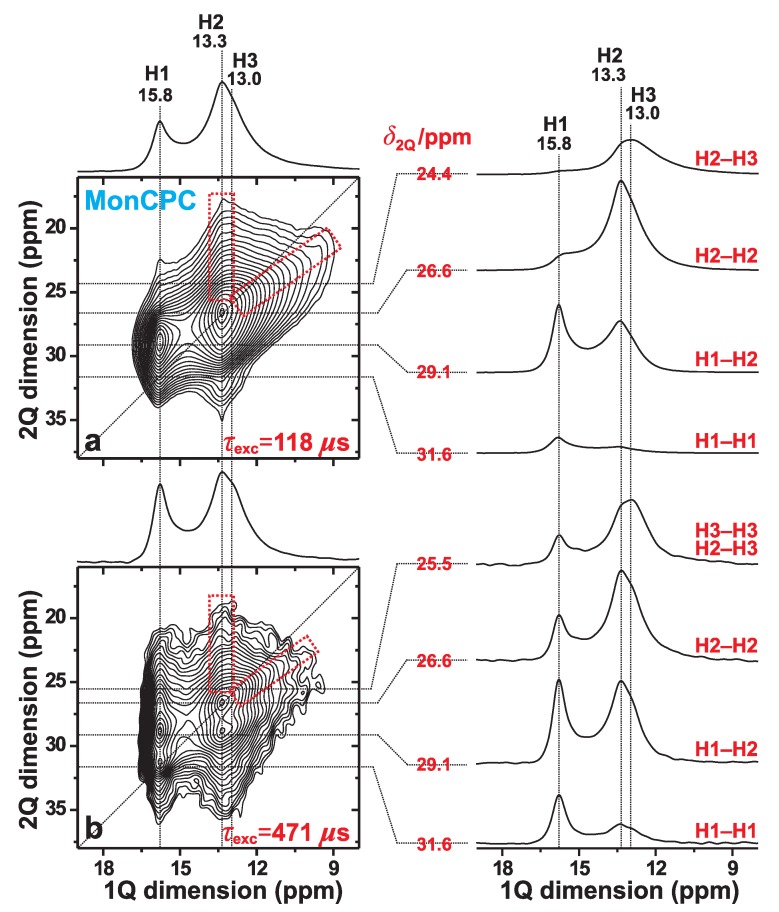
2Q–1Q correlation ${}^{1}$H NMR spectra (left panel) recorded from the MonCPC cement at 34.00 kHz MAS and 9.4 T, using the (**a**) [*S*R2${}_{2}^{1}$] (ref. [26]) and (**b**) [*S*R2${}_{4}^{1}$] (ref. [27]) pulse sequences for 2QC excitation periods of (**a**) 118 μs and (**b**) 471 μs. The horizontal 1Q projection is displayed at the top of the 2D NMR spectrum. The NMR-peak assignments to the H1, H2, and H3 sites of monetite are indicated at the top. The right panel displays slices along the 1Q dimension, extracted at the as-indicated 2Q shifts for the 2Q(H*m*–H*n*) correlations identified to the right of each slice. The lowest contour level is set at 3% of the maximum 2D NMR peak amplitude. The dashed red rectangles indicate the signal regions associated with the ${}^{1}$H2–${}^{1}$H3 2Q–1Q correlations observed in both 2D NMR spectra.

Figure 4 displays 2Q–1Q ${}^{1}$H NMR spectra recorded at 9.4 T and 34.00 kHz MAS from the MonCPC cement. They reveal a markedly worse spectral resolution relative to that of the monetite standard of Figure 3; the degraded resolution partially stems from ${}^{1}$H NMR-peak broadenings associated with the lower MAS rate employed (see Figure 2), but also from the emphasized structural disorder of the “monetite-like” phase in the CPC. Nonetheless, the results observed are of sufficient quality to conclude the absence of any fundamental difference in the overall structural feature among the two monetite phases (also see Section 3.4.3).

As expected for a short 2QC excitation interval of $\tau_{\mathrm{rec}}=118$ μs, the two 2Q–1Q NMR peaks associated with the H1–H2 and H2–H2 pairs dominate the 2D NMR spectrum, while the signals from the longer-range H3–H3 and H1–H1 pairs are absent and very weak, respectively (Figure 4a). In contrast, at the longer excitation period of $\tau_{\mathrm{rec}}=471$ μs, numerous 2Q–1Q correlations are observed (Figure 4b). The ${}^{1}$H1–${}^{1}$H1 autocorrelation signal is well-developed at its expected 2D NMR coordinate {$\delta_{2Q}$, $\delta_{1Q}$} = {31.6, 15.8} ppm. Moreover, while the spectral resolution at 34.00 kHz MAS does not permit unambiguous identification of the expected H3–H3 autocorrelation, its presence is strongly suggested from the enhanced NMR-signal intensity observed ≈13 ppm in the slice shown in the right panel of Figure 4b ($\delta_{2Q}=25.5$ ppm) relative to that observed in Figure 4a ($\delta_{2Q}=24.4$ ppm). We also comment that the pronounced resonance-spread towards lower $\delta_{2Q}$ values observed from the ${}^{1}$H1 resonance at $\delta_{H}=15.8$ ppm in the 1Q dimension of the 2Q–1Q NMR spectrum in Figure 4b stems from significant contributions from “indirect 2QC signals” due to the long 2QC excitation interval of 471 μs (see Section 3.2). Nonetheless, those signals are negligible in the NMR spectrum recorded at $\tau_{\mathrm{exc}}=118$ μs (Figure 4a).

To summarize, the 2Q–1Q NMR spectra recorded from the MonCPC cement suggest that its monetite-like phase overall shares the same set of proton–proton contacts as those of the phase-pure monetite sample. The latter was furthermore shown to be consistent with the neutron-diffraction-derived structure of monetite [6] (see Section 3.2).

### 3.4. ${}^{1}$H–${}^{31}$P Proximities in Monetite: ${}^{31}$P{${}^{1}$H} HETCOR NMR

#### 3.4.1. Relative ${}^{1}$H–${}^{31}$P Contacts

${}^{1}$H$\to^{31}$P CP relies on dipolar-coupling-mediated magnetization transfers from ${}^{1}$H sites nearby a ${}^{31}$PO${}_{4}$ group [42], where the dipolar coupling constant ($b_{\mathrm{HP}}$; in Hz) relates to the internuclear distance $r_{\mathrm{HP}}$ by

(2) $$b_{\mathrm{HP}}=Kr_{\mathrm{HP}}^{-3},\;\mathrm{with}\;K=-\mu_{0}\gamma_{H}\gamma_{P}\hbar /8\pi^{2},$$

where $\gamma_{H}$ and $\gamma_{P}$ denote the magnetogyric ratio of ${}^{1}$H and ${}^{31}$P, respectively, and $\mu_{0}$ is the permeability of vacuum [21,22,23]. In practice, ${}^{1}$H–${}^{31}$P dipolar interactions offer the possibility to probe internuclear distances within ≲1 nm. CP leads to the sole detection of ${}^{31}$P nuclei in close proximity to *some*${}^{1}$H sites in the structure. However, just as 2Q–1Q ${}^{1}$H correlation NMR reveals the closest spatial proximities among proton-pairs in the structure by exploiting homonuclear ${}^{1}$H–${}^{1}$H interactions (see Section 3.2), the CP-based ${}^{31}$P{${}^{1}$H} HETCOR NMR experiment informs about *which* ${}^{1}$H and ${}^{31}$PO${}_{4}$ groups that are closest neighbors. Here, a 2D NMR correlation peak appearing at the spectral coordinate {$\delta_{H}$, $\delta_{P}$} evidences that the corresponding ${}^{1}$H and ${}^{31}$P structural sites (that resonate at $\delta_{H}$ and $\delta_{P}$, respectively) are in close proximity [34], where the chemical shifts of ${}^{31}$P and ${}^{1}$H are encoded along the horizontal and vertical dimensions of the 2D NMR spectrum, respectively.

**Figure 5:**
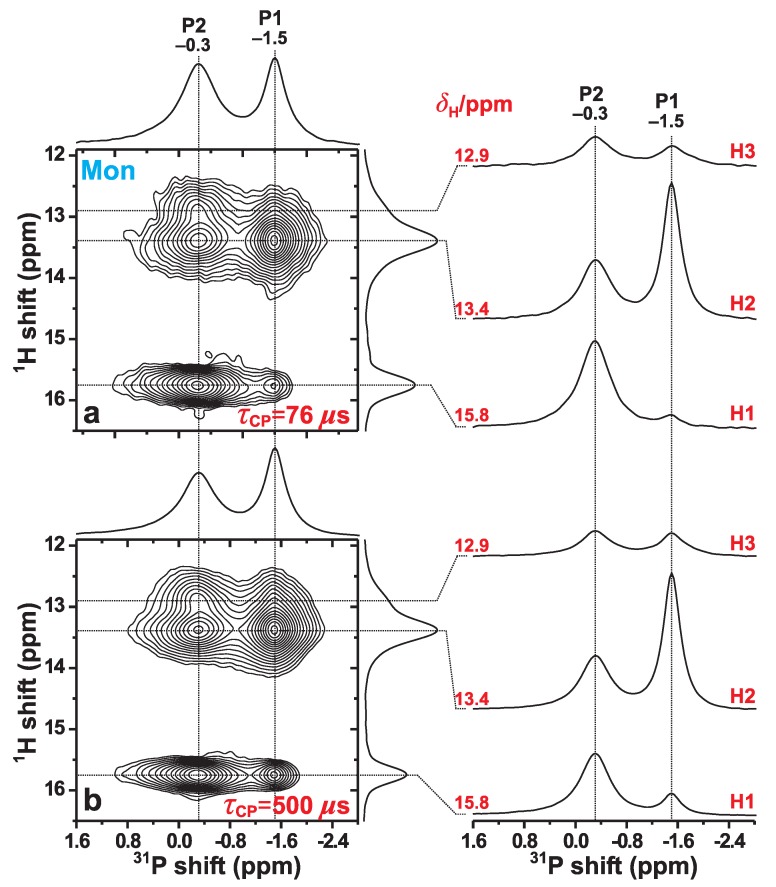
${}^{31}$P{${}^{1}$H} HETCOR NMR spectra, obtained from the Mon specimen at 66.00 kHz MAS and $B_{0}=9.4$ T, using ${}^{1}$H$\to^{31}$P CP contact time-periods of (**a**) $\tau_{\mathrm{CP}}=76$ μs and (**b**) $\tau_{\mathrm{CP}}=500$ μs. Each 2D NMR spectrum is shown together with projections along the ${}^{31}$P (horizontal; top) and ${}^{1}$H (vertical; right) spectral dimensions. The right panel displays slices along the ${}^{31}$P dimension, extracted at the as-indicated ${}^{1}$H chemical shifts ($\delta_{H}$).

We now examine the ${}^{31}$P{${}^{1}$H} HETCOR NMR results obtained from the Mon specimen that are shown in Figure 5. They were acquired at 66 kHz MAS for two distinct CP contact intervals of 76 μs and 500 μs. In the 2D NMR spectrum recorded with the *shortest* contact period (Figure 5a), the two most intense 2D NMR peaks are observed at the {$\delta_{H}$, $\delta_{P}$} coordinates {13.4, −1.5} ppm and {15.8, −0.3} ppm. They stem from the H2–P1 and H1–P2 pairs, respectively, where the former involves the H–P contact within an HPO${}_{4}^{2-}$ group, whereas the H1–P2 distance is unusually short owing to the geometry of the P2–O–H1–O–P2 structural fragment; see Figure 1 and Table 2. Then while the correlations involving P2 and each of H2 and H3 reveal moderately large intensities, those between P1 and each of H1 and H3 are very weak, as expected from the absence of any direct bonds between the latter protons and the P1-centered phosphate groups (Figure 1).

For HETCOR experimentation with short contact periods ($\tau_{\mathrm{CP}}≲100$ μs), the integrated 2D NMR peak intensity centered at the coordinate {$\delta_{H}$, $\delta_{P}$} is proportional to the square of the heteronuclear dipolar coupling constant ($b_{\mathrm{HP}}^{2}$) associated with the ${}^{1}$H–${}^{31}$P pair [23] (see the discussion in Section 3.2). Note that ${}^{1}$H spin diffusion during CP is strongly suppressed by the fast MAS (66 kHz) and is not expected to affect any 2D NMR peak intensity for the short contact period $\tau_{\mathrm{CP}}=76$ μs. We obtained the integrated 2D NMR-peak intensity, I(H*m*–Pn), associated with each ${}^{1}$H–${}^{31}$P pair among the P1/P2 and H1/H2/H3 sites. This required deconvolution of the two heavily overlapping ${}^{1}$H2 and ${}^{1}$H3 resonances in Figure 5a, which was performed with the DMFit software [43]. Then, each *fractional* 2D NMR intensity was calculated according to

(3) $$f_{\mathrm{NMR}}\left(Hm-Pn\right)=I\left(Hm-Pn\right)/I_{\mathrm{tot}},$$

where the total signal intensity ($I_{\mathrm{tot}}$) is given by the sum over the contributions from the six distinct H*m*–P*n* pairs with *m* = {1, 2, 3} and *n* = {1, 2}. The {$f_{\mathrm{NMR}}($H*m*–Pn)} data are presented in Table 3, along with the corresponding {$f_{\mathrm{ND}}($H*m*–Pn)} results calculated from the diffraction-derived crystal structure [6]. Here each $f_{\mathrm{ND}}($H*m*–Pn) value was obtained from the corresponding squared effective dipolar-coupling constant $b_{\mathrm{eff}}^{2}($H*m*–Pn) [defined analogously with Equation (1)] according to

(4) $$f_{\mathrm{ND}}\left(Hm-Pn\right)=b_{\mathrm{eff}}^{2}\left(Hm-Pn\right)/b_{\mathrm{eff}}^{2}\left(\mathrm{tot}\right),$$

where $b_{\mathrm{eff}}^{2}($tot) is the sum over the contributions from all proton pairs:

(5) $$b_{\mathrm{eff}}^{2}\left(\mathrm{tot}\right)=\sum_{m=1,2,3}\sum_{n=1,2}b_{\mathrm{eff}}^{2}\left(Hm-Pn\right).$$

Each NMR [$f_{\mathrm{NMR}}($H*m*–Pn)] and neutron diffraction [$f_{\mathrm{ND}}($H*m*–Pn)] derived entity conveys the relative H*m*–P*n* contacts. Those obtained by NMR relate roughly as follows (Table 3):

(6) H2−P1>H1−P2>H3−P2≳H2−P2≫H3−P1>H1−P1,

while their $f_{\mathrm{ND}}($H*m*–Pn) counterparts reveal the following very close trend:

(7) H2−P1≈H1−P2>H3−P2>H2−P2≫H3−P1≈H1−P1.

Notwithstanding minor quantitative discrepancies that are discussed below, the overall good agreement between the monetite structure reported by Catti et al. [6] and the ${}^{31}$P{${}^{1}$H} HETCOR results of Figure 5a is gratifying, including their excellent qualitative mutual agreement concerning the weakest ${}^{1}$H–${}^{31}$P contact in monetite, i.e., the H1–P1 pair (Table 3). The two shortest H1–P1 distances are 382 pm and 389 pm (Table 2), which are markedly longer than for any other ${}^{1}$H–${}^{31}$P pair [6]. This is indeed mirrored by a very weak 2D NMR correlation signal observed at the coordinate {15.8, –1.5} ppm. Yet, for the longer contact period of $\tau_{\mathrm{CP}}=500$ μs, this 2D NMR peak is markedly stronger (Figure 5b), as is that from the second weakest ${}^{1}$H–${}^{31}$P contact, i.e., H3–P1 (Table 2). The slower ${}^{1}$H → ${}^{31}$P magnetization transfers within these two ${}^{1}$H–${}^{31}$P pairs reflect their longer interatomic distances.

#### 3.4.2. Effective H–P Distances

To reach a physically more intuitive picture about the agreement and derivations between the present NMR results and the crystal structure of ref. [6], we converted each $f_{\mathrm{NMR}}($H*m*–Pn) value into an “*effective*” interatomic distance, $r_{\mathrm{eff}}($H*m*–Pn). For the neutron-diffraction derived structure, the set of distances was calculated from the squared effective dipolar-coupling constants {$b_{\mathrm{eff}}^{2}($H*m*–Pn)} obtained from the distances presented in Table 2 and using the expression

(8) $$r_{\mathrm{eff}}\left(Hm-Pn\right)={\left(\frac{MK^{2}}{b_{\mathrm{eff}}^{2}\left(Hm-Pn\right)}\right)}^{1/6},$$

where *K* is defined in Equation (2). However, the ${}^{31}$P{${}^{1}$H} HETCOR NMR spectrum of Figure 5a *alone* does not admit determining $r_{\mathrm{eff}}($H*m*–Pn). Yet, since the set of {$f_{\mathrm{NMR}}($H*m*–Pn)} data comprises complete information about the *relative* H*m*–P*n* proximities, knowledge of *one* distance is sufficient to determine all others [21]. However, all six H*m*–P*n* distances are reported in ref. [6] for the present case of the monetite structure (Table 2). Hence, a more accurate and less biased option is to assume that the total integrated NMR-signal intensity [$I_{\mathrm{tot}}$; see Equation (3)] in the HETCOR spectrum of Figure 5a may be equated with $b_{\mathrm{eff}}^{2}($tot) calculated from the crystal structure *via* Equation (5). Then, the NMR-derived squared effective dipolar coupling constant of each H*m*–P*n* pair may be calculated from $f_{\mathrm{NMR}}($H*m*–P$n)\cdot b_{\mathrm{eff}}^{2}($tot) and converted into an effective H*m*–P*n* distance, $r_{\mathrm{eff}}^{\mathrm{NMR}}($H*m*–Pn), by using Equation (8).

Table 3 lists the resulting $r_{\mathrm{eff}}^{\mathrm{NMR}}$ and $r_{\mathrm{eff}}^{\mathrm{ND}}$ results for each of the six distinct H–P pairs. Except for H1–P1 and H3–P1, the agreement between the NMR and neutron diffraction results is very good (<8 pm deviation, i.e., ⩽3% relative discrepancy). It is not surprising that the largest deviations between the $r_{\mathrm{eff}}^{\mathrm{NMR}}$ and $r_{\mathrm{eff}}^{\mathrm{ND}}$ distances are observed for the two weakest H–P contacts in the monetite structure. For the H1–P1 pair, the NMR-derived effective distance is 18 pm shorter than its neutron-diffraction counterpart, whereas for the H3–P1 pair, it is 72 pm shorter. These discrepancies may either reflect experimental uncertainties associated with these longest H–P distances, or that the precise H1 and H3 positions of the crystal structure of ref. [6] may be in slight error. The difficulties in locating the precise proton positions by diffraction are well known (despite using neutrons); indeed, previous NMR reports have highlighted similar discrepancies to diffraction-derived calcium phosphate structures [44,45,46].

Concerning the (minor) deviations among the shorter effective distances (i.e., stronger H–P contacts), the $r_{\mathrm{eff}}^{\mathrm{ND}}$ values are equal for the H1–P2 and H2–P1 pairs (Table 3), while the NMR results yield $r_{\mathrm{eff}}^{\mathrm{NMR}}($H1–P2$)>r_{\mathrm{eff}}^{\mathrm{NMR}}($H2–P1). This is reflected in the (quantitative) ranking of their relative contacts in Equations (6) and (7). These subtle discrepancies may be traced to the dipolar-coupling topology of the proton sites in monetite. The two *shortest* H2–P1 distances are 225 pm and 228 pm, yet there are two additional (longer) distances of 358 pm and 360 pm, which are nevertheless shorter than those of the H1–P1 and H3–P1 pairs. A similar situation applies to the H2–P2 contacts, which involves two additional contacts at 329 pm. Once those H2–P1 and H2–P2 pairs are also accounted for, an excellent agreement is observed between the NMR and neutron diffraction results for all H*m*–P*n* pairs (except for H1–P1 and H3–P1): both techniques lead to the relative H*m*–P*n* contacts given by Equation (6), while the $r_{\mathrm{eff}}^{\mathrm{NMR}}$ and $r_{\mathrm{eff}}^{\mathrm{ND}}$ data agree within 5 pm.

#### 3.4.3. Discussion on the H–P Contacts in Monetite and MonCPC

Once concluding a very good (overall) quantitative accordance between the present NMR results and the neutron-diffraction derived monetite structure, we summarize some main inferences in relation to the structural fragment shown in Figure 1, which is based on the atom coordinates of ref. [6]. The H2–P1 and H1–P2 pairs exhibit the shortest interatomic distances in monetite, in the case of H2–P1 because they are constituents of an HPO${}_{4}^{2-}$ group, whereas the H1–P2 distance becomes comparatively short due to the geometry around the P2–O–H1–O–P2 linkages. The H3–P2 and H2–P2 pairs reveal the second strongest H–P contacts, and thereby second shortest effective interatomic distances; see Table 3. These pairs may be attributed to involve hydrogen bonds between each H1/H3 proton and the O atom of a P2 phosphate tetrahedron. The third group of H–P contacts concerns H3–P1 and H1–P1, which are both much weaker than the others because they neither belong to the same HPO${}_{4}^{2-}$ moiety nor involve hydrogen bonds.

The results of Table 3 also provide some hints of the nature of the very similar—yet distinct—contacts between the H1 and H3 protons and the P2${}_{A}$ and P2${}_{B}$ sites that alternate along the chain of HPO${}_{4}^{2-}$ tetrahedra in Figure 1. While the H3 protons constitute the acidic proton of the P2${}_{A}$-centered HPO${}_{4}^{2-}$ groups, the nature of the H1–P2 contacts are less obvious. Yet, *if* all H1 protons are identified as participating in hydrogen bonds to all P2${}_{A}$/P2${}_{B}$ phosphate groups, clues to the failure of ${}^{31}$P MAS NMR to resolve their resonances (Figure 1a) are given: one O atom of the P2${}_{B}$-centered tetrahedron involves a (primarily) covalent bond to H3, whereas another forms a hydrogen bond to H1 (Figure 1). In contrast, the P2${}_{A}$ phosphate group forms hydrogen bonds to *both* H1 and H3. These subtle differences in the P2${}_{A}$ and P2${}_{B}$ contacts with the H1 and H3 protons naturally explain that whereas the P2${}_{A}$ and P2${}_{B}$ sites exhibit slightly different chemical shifts, they remain sufficiently close to merely produce a peak-broadening of the net ${}^{31}$P2 resonance (Figure 1a).

According to ref. [6], the H1 site is positioned centrosymmetrically between the P2${}_{A}$ and P2${}_{B}$ atoms, whereas the H3 protons are distributed among two close but distinct positions. Hence, the disorder of the latter is “static” rather than “dynamic”. While neither ref. [6] nor our present results may preclude the presence of H mobility (dynamic disorder), both suggest static disorder of the H3 sites. Notably, as follows from Section 2.2 and Figure 2b, there are no indications of any temperature dependence of the ${}^{1}$H NMR chemical shifts (as could be expected in the case of proton mobility). Yet, the temperature elevation due to frictional heating among the NMR experiments involving the three MAS probeheads and spinning speeds is only estimated to be ≈20 ${}^{\circ }$C higher in the 2.5 mm (≈62 ${}^{\circ }$C [47]) and 1.3 mm (≈65 ${}^{\circ }$C, calibrated in our laboratory) rotors relative to the 4 mm counterpart (≈44 ${}^{\circ }$C [48]). Here each stated temperature is that of the center of the sample for a nominal ambient temperature of 25 ${}^{\circ }$C.

We next contrast the inference from the present ${}^{1}$H{${}^{31}$P} HETCOR NMR results with the HETCOR spectrum obtained from the MonCPC specimen shown in Figure 8a of Yu et al. [32]. That was recorded at $B_{0}=9.4$ T and a lower MAS rate of 34 kHz, which coupled with the emphasized structural disorder of its monetite phase (see Section 3.3) and the presence of NMR signals from additional phases in the cement lead to lower spectral resolution. Nonetheless, while the compromised spectral resolution did not permit resolving the ${}^{1}$H2 and ${}^{1}$H3 resonances [32], the monetite-stemming 2D NMR correlation signals observed for a (short) contact period of $\tau_{\mathrm{CP}}=118$ μs accord qualitatively with those of Figure 5a: as is most transparent from the slices along the ${}^{31}$P spectral dimension that were taken at $\delta_{H}=15.8$ ppm (H1) and $\delta_{H}=13.3$ ppm (H2) in Figure 8a of ref. [32], the relative H*m*–P*n* contacts decrease according to

(9) H2−P1>H1−P2≈H2−P2>H1−P1.

This order is in excellent semiquantitative agreement with that concluded for the Mon structure [Equation (6)]. The sole qualitative difference concerns the relative contacts in the H1–P2 and H2–P2 pairs. Yet, Table 3 reveals only minor differences in their respective $r_{\mathrm{eff}}^{\mathrm{NMR}}$ values, and the *apparent* discrepancies among the HETCOR NMR results from the monetite phases of the Mon and MonCPC specimens are readily rationalized from the slightly longer contact period ($\tau_{\mathrm{CP}}=118$ μs) employed in ref. [32]. As is evident by comparing the two HETCOR spectra of Figure 5a, lengthening of $\tau_{\mathrm{CP}}$ enhances the signal intensities from ${}^{1}$H–${}^{31}$P pairs with longer distances so that they become comparable to the intensities observed from the shorter ones. To summarize, the various H*m*–P*n* contacts in the monetite component of MonCPC are overall very similar to those of the more ordered Mon structure.

### 3.5. Interatomic-Distance Determination Procedure

The extraction of accurate homonuclear (e.g., ${}^{1}$H–${}^{1}$H) or heteronuclear (e.g., ${}^{1}$H–${}^{31}$P) internuclear distances from multi-spin systems is generally performed by recording a series of 2D NMR experiments with progressively increasing dipolar recoupling intervals [21,22,23,49,50], e.g., the $\tau_{\mathrm{CP}}$ and $\tau_{\mathrm{exc}}$ period for the respective HETCOR and 2Q–1Q correlation protocol. Besides the time-consuming process to arrange such a series of 2D NMR data-sets, the distance-analysis generally requires assistance by fitting to numerically exact simulations [49,50]. While fairly straightforward for heteronuclear systems, the procedure easily becomes painstaking for homonuclear cases due to their multi-spin character, unless approximations/assumptions are made. Moreover, the accuracy of numerical simulations is compromised for NMR analyses of structurally disordered inorganic phosphate phases, such as monetite. Numerically exact simulations may be avoided if knowledge about an “effective” H–P (or H–H) distance is sufficient for each H–P (H–H) pair, which is attainable from a series of 2D NMR experiments by fitting the initial signal-buildup to obtain a dipolar second moment [25].

Here the present protocol for obtaining effective H–P distances from one sole ${}^{31}$P{${}^{1}$H} HETCOR NMR experiment offers an attractive alternative. Yet, it should be stressed that its implementation requires information about (at least) one H–P distance in the structure, from which all others may be derived from the set of integrated 2D NMR intensities observed from the ${}^{1}$H–${}^{31}$P pairs (see Section 3.4). This idea is certainly not new, e.g., see Schnell and Spiess [21], yet we are not aware of much concrete applications of this comparatively straightforward approach. Its distance-analysis strategy is generally applicable to any combination of spins, also encompassing homonuclear systems. Although it could not be performed on our 2Q–1Q ${}^{1}$H NMR spectra due to too extensive overlap between the ${}^{1}$H2 and ${}^{1}$H3 resonances, it is perfectly applicable in scenarios where the various correlation NMR signals are readily resolved; indeed, once grouping together the H2 and H3 structural sites (“H23”) and their accompanying NMR signals, a very good agreement was observed among the three H1–H1, H1–H23, and H23–H23 interatomic contacts derived by NMR and those of neutron diffraction.

## 4. Concluding Remarks

From high speed ${}^{1}$H NMR experiments at 34 kHz or 66 kHz MAS, all three ${}^{1}$H resonances from the crystallographically inequivalent H1, H2, and H3 sites of monetite were resolved at {15.8, 13.4, 12.9} ppm, respectively; the assignment was further confirmed by 2Q–1Q correlation NMR experiments. This appears to be the first *unambiguous* ${}^{1}$H NMR-peak assignment of the H2 and H3 sites of monetite. These results confirm the previous tentative assignment made by Xue and Kanzaki from an NMR spectrum with inferior resolution [19]. Moreover, the NMR-derived relative ${}^{1}$H–${}^{1}$H proximities among the {H1, H2, H3} sites of monetite accorded very well with those reported earlier from a neutron diffraction study [6]. The overall shortest distances are observed for the ${}^{1}$H2–${}^{1}$H2 sites (323 pm), followed by those of H2–H3 (356 pm) and H1–H2 (376 pm), whereas the shortest distances among the H1–H1 and H3–H3 sites are markedly longer (663 pm) because they are separated by one unit-cell length (see Table 1).

From the integrated 2D NMR intensities of the resolved H*m*–P*n* signals in a ${}^{31}$P{${}^{1}$H} HETCOR NMR spectrum acquired with a short contact period at 66 kHz MAS, we derived the effective (average) distance within each of the six pairs of {P1, P2} and {H1, H2, H3} sites. This was achieved by utilizing one single 2D NMR spectrum from which all relative H–P contacts were extracted. Yet, to convert these results into average interatomic distances, we assumed that the total integrated HETCOR NMR intensity is equal to the sum of squared dipolar-coupling constants calculated from a neutron-derived crystal structure of monetite [6]. The thereby NMR-derived set of six average P1/P2–H1/H2/H3 distances agreed very well with those of ref. [6]. Notably, this distance-determination strategy is generally applicable to any combination of spins, including homonuclear systems, provided that one interatomic distance in the structure is known, and from which all others are derived by using the relative integrated 2D NMR signal intensities.

The relative H*m*–P*n* contacts in the monetite structure fall into three groups (see Figure 1 and Table 3): the shortest distance involves the acidic proton (H2) of the (H2)(P1)O${}_{4}^{2-}$ tetrahedron (224 pm), for which the H2–O bond is primarily of covalent character. The second shortest H1–P2 distance (233 pm), on the other hand, becomes short due to the geometry around the P2${}_{A}$–O–H1–O–P2${}_{B}$ fragment, where the two H1–O bond lengths are intermediate of a those typical for covalent and hydrogen bonds. Slightly longer (average) H–P distances are encountered for the H2–P2 (246 pm) and H3–P2 (243 pm) pairs, where H3 is the acidic proton of the (H3)(P2)O${}_{4}^{2-}$ tetrahedron, while H2–P2 constitutes a hydrogen bond. Finally, significantly longer average H–P distances of 294 pm and 367 pm are observed for the H3–P1 and H1–P1 pairs, which do not involve any direct bonds between the protons and the phosphate groups.

The two P2${}_{A}$ and P2${}_{B}$ centered phosphate groups alternate with the H1 and H3 protons (Figure 1), where the latter site is disordered as it may appear at two slightly different positions [6]. Hence, while the ${}^{31}$P2${}_{A}$ and ${}^{31}$P2${}_{B}$ environments are very similar, they differ in their contacts with H1 and H3. Here the P2${}_{B}$ phosphate moiety involve one covalent bond to its acidic proton H3, along with one hydrogen bond to H1. The P2${}_{A}$ group, on the other hand, merely forms two hydrogen bonds to each of H1 and H3, however, with a comparatively short H1⋯P2${}_{A}$ distance. We propose that these very subtle bonding differences between the P2${}_{A}$ and P2${}_{B}$ tetrahedra and their surrounding H1 and H3 protons account for the inability of ${}^{31}$P MAS NMR to resolve their resonances (in the present study, as well as in previous reports [12,13,15,16,17,18,20]). Yet, the very minor chemical-shift differences rationalize the markedly broader ${}^{31}$P NMR peak observed from the P2${}_{A}$/P2${}_{B}$ sites relative to their P1 counterpart.

Moreover, 2Q–1Q ${}^{1}$H NMR experimentation performed on the monetite-based and pyrophosphate-bearing CPC sample (MonCPC) revealed overall similar H–H contacts in its monetite component as that found for the phase-pure monetite structure. Similarly, the ${}^{31}$P{${}^{1}$H} HETCOR NMR results of the latter accorded with those presented previously from the MonCPC sample in ref. [32]. Altogether, these observations suggest an overall intact monetite structure in the cement, albeit it is more disordered.

## Figures and Tables

**Table 1:** Shortest H–H Interatomic Distances (in pm) in Monetite. ^a^

Site	H1	H2	H3
H1	663	376;377;399	435;440
H2		323;357	356;363;374
H3			663

^a^ Only the *shortest* r(H*m*–Hn) distances are listed, where multiple values reflects the multiplicity (*M*) of the given H*m*–H*n* contact. The H atom coordinates were obtained from the neutron diffraction results of Catti et al. [6].

**Table 2:** Shortest H–P Interatomic Distances (in pm) in Monetite. ^a^

Site	H1	H2	H3
P1	382;389	225;228	347;396
P2	222;230	251;251	233;238

^a^ Only the *shortest* r(H*m*–Pn) distances are listed, where the number of entries for a given atom-pair reflects its multiplicity *M*. The H and P atom coordinates were obtained from Catti et al. [6].

**Table 3:** Effective H–P Distances in Monetite as Deduced from NMR and Neutron Diffraction. ^a^

Site	H1		H2		H3	
	$f_{\mathrm{NMR}}\left(f_{\mathrm{ND}}\right)$	$r_{\mathrm{eff}}^{\mathrm{NMR}}\left(r_{\mathrm{eff}}^{\mathrm{ND}}\right)$	$f_{\mathrm{NMR}}\left(f_{\mathrm{ND}}\right)$	$r_{\mathrm{eff}}^{\mathrm{NMR}}\left(r_{\mathrm{eff}}^{\mathrm{ND}}\right)$	$f_{\mathrm{NMR}}\left(f_{\mathrm{ND}}\right)$	$r_{\mathrm{eff}}^{\mathrm{NMR}}\left(r_{\mathrm{eff}}^{\mathrm{ND}}\right)$
P1	0.016(0.012)	367(385)	0.312(0.290)	224(226)	0.061(0.016)	294(366)
P2	0.246(0.296)	233(226)	0.176(0.156)	246(251)	0.189(0.230)	243(235)

^a^$f_{\mathrm{NMR}}($H*m*–Pn) and $f_{\mathrm{ND}}($H*m*–Pn) represent the relative degree of H*m*–P*n* interatomic contact as obtained from the ${}^{31}$P{${}^{1}$H} HETCOR NMR spectrum of Figure 5 and by neutron diffraction [6], respectively, using the corresponding Equations (3) and (4). The 1σ uncertainties are 0.017 and 0.006 for $f_{\mathrm{NMR}}$ and $f_{\mathrm{ND}}$, respectively. $r_{\mathrm{eff}}^{\mathrm{NMR}}($H*m*–Pn) and $r_{\mathrm{eff}}^{\mathrm{ND}}($H*m*–Pn) denote the respective “effective” H*m*–P*n* interatomic distances, which were calculated from Equation (8) using the set of H*m*–P*n* distances listed in Table 2; the 1σ uncertainties of $r_{\mathrm{eff}}^{\mathrm{NMR}}$ and $r_{\mathrm{eff}}^{\mathrm{ND}}$ are 6 pm and 1 pm, respectively.

## Supplementary Materials

The following are available online at https://www.mdpi.com/1422-0067/20/24/6356/s1, Figure S1: Deconvoluted ${}^{31}$P and ${}^{1}$H MAS NMR spectra recorded from the Mon sample.
