# Supplementary material for: The Monetite Structure Probed by Advanced Solid-State NMR Experimentation at Fast Magic-Angle Spinning
Source: Int J Mol Sci. 2019 Dec 17;20(24):6356. doi: 10.3390/ijms20246356 (PMC6940740; doi:10.3390/ijms20246356)
Supplement: Supplementary file 1 [file ijms-20-06356-s001.pdf]

Supporting Information for
The Monetite Structure Probed by Advanced Solid-State NMR Experimentation at Fast Magic-Angle Spinning

Yang Yu[1], Baltzar Stevensson[1], Michael Pujari-Palmer[2], Hua Guo[1], Håkan Engqvist[2], and Mattias Edén[1,*]

[1]Department of Materials and Environmental Chemistry, Stockholm University, SE-106 91 Stockholm, Sweden
[2]Applied Material Science, Department of Engineering, Uppsala University, SE-751 21 Uppsala, Sweden

[*]Corresponding author. E-mail: *mattias.eden@mmk.su.se*

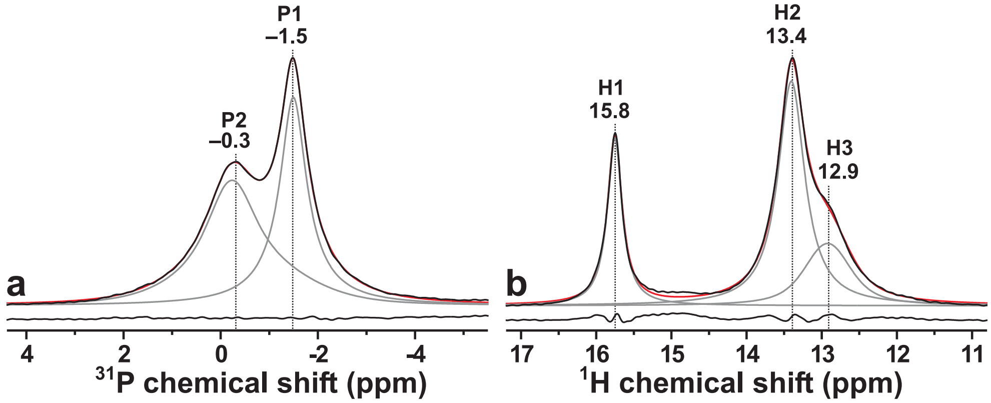

Figure S1. Deconvoluted (a) ^{31}P and (b) ^{1}H MAS NMR spectra (obtained at $\nu_r = 66$ kHz for ^{1}H). The experimental NMR spectra (black traces) are shown together with the best-fit result (red traces), as well as the component NMR peaks (grey traces). The curve beneath each NMR spectrum represents the difference between the experiment and best-fit. Note that each ^{31}P resonance (grey traces) from the respective P1 and P2 sites in (a) represents the sum of two signals: one narrow (fitted with the constraint FWHM<1.5 ppm) and one broad (FWHM<4.2 ppm) NMR peak, which were necessary to invoke to emulate the structural disorder of the monetite structure. We refer to refs. [S1] and [S2] for details about the deconvolution procedure. Each of the three ^{1}H NMR resonances from the ^{1}H1, ^{1}H2, and ^{1}H3 sites in (b) were accounted for by one peak.

References

[S1] Yu, Y.; Guo, H.; Pujari-Palmer, M.; Stevensson, B.; Grins, J.; Engqvist, H.; Edén, M. Advanced Solid-State ^{1}H/^{31}P NMR Characterization of Pyrophosphate-Doped Calcium Phosphate Cements for Biomedical Applications: The Structural Role of Pyrophosphate. *Ceram. Int.* **2019**, *45*, 20642–20655.

[S2] Guo, H.; Pujari-Palmer, M.; Yu, Y.; Stevensson, B.; Engqvist, H.; Edén, M. Quantitative Phase Analyses of Pyrophosphate-Bearing Monetite and Brushite Biocements by Solid-State NMR and Powder XRD. *Ceram. Int.* **2019**, submitted.
